# Supplementary material for: FAPM: functional annotation of proteins using multimodal models beyond structural modeling
Source: Bioinformatics. 2024 Nov 14;40(12):btae680. doi: 10.1093/bioinformatics/btae680 (PMC11630832; doi:10.1093/bioinformatics/btae680)
Supplement: btae680_Supplementary_Data [file btae680_supplementary_data.zip › FAPM-Supplementary.docx]

# Supplementary

| Name | location | Functional labels |
| --- | --- | --- |
| Gp41 | cytosol | DNA binding; protein-protein interaction |
| Gp44 | cytosol | DNA binding |
| Gp45 | cytosol | DNA binding |
| Gp46 | cytosol | Protein-protein interaction |
| Gp49 | cytosol | RNA binding |
| Gp60 | cytosol | Protein-protein interaction |
| Gp33 | cytosol | Protein-protein interaction |
| Gp27 | cytosol | DNA binding |
| Gp35.1 | cytosol | Toxin activity |
| RpbA | cytosol | Protein-protein interaction |
| Exod | cytosol | Dual nuclease activity |
| Gp57B | cytosol | Catalase activity |
| Pin | cytosol | Peptidase inhibitor activity; Protein-protein interaction |
| Mrh | cytosol | DNA binding; Protein-protein interaction |
| Cef | cytosol | RNA binding; Protein-protein interaction |
| MsyB | cytosol | Protein-protein interaction, E. coli |
| YciZ | cytosol | Signal transduction; Protein-protein interaction, B. subtilis |
| YlaB | inner membrane | Signal transduction; Protein-protein interaction, B. subtilis |
| Ysdb | inner membrane | Signal transduction; Protein-protein interaction, B. subtilis |
| RsiW | transmembrane | Signal transduction; Protein-protein interaction, B. subtilis |
| Gp2 | cytosol | Protein-protein interaction |
| Gp6 | cytosol | DNA binding |
| Gp8 | cytosol | DNA binding |
| Gp12 | cytosol | DNA binding |
| ss1 |  | DNA binding |
| sm2 | cytosol | Protein-protein interaction |
| sl1 | cytosol | Protein-protein interaction |
| 75 | cytosol | DNA binding |
| 235 | cytosol | DNA binding |

**Supplementary Table S1** the experimentally verified bacteriophage and bacteria protein that are unannotated in UniProt. Proteins are bacteriophage protein unless listed otherwise.

## Impact of different prompts

We selected four frequently occurring species from our dataset: Homo, Mus, Arabidopsis, and Saccharomyces. Among them, Homo and Mus are classified as mammals, Arabidopsis as a plant, and Saccharomyces as a fungus. For the same set of protein sequences, we analyzed the prediction differences when provided these four species as prompt. Figure S1 illustrates the distribution differences in predictions for two protein functions. Generally, fungi do not possess an immune system, therefore, proteins in fungi are unlikely to have an "immune response" function. Accordingly, when the prompt is Saccharomyces, the probability predicted by FAPM for this function is significantly lower than for the other three prompts in Figure S1. Another function showed in Figure S1 is "skeletal system development". The skeleton serves as the bony framework of the body in vertebrates (endoskeleton) or the hard outer envelope of insects (exoskeleton or dermoskeleton). As such, proteins in Arabidopsis and Saccharomyces cannot have this function, and the probabilities for this function given by FAPM is almost zero given these two prompts.


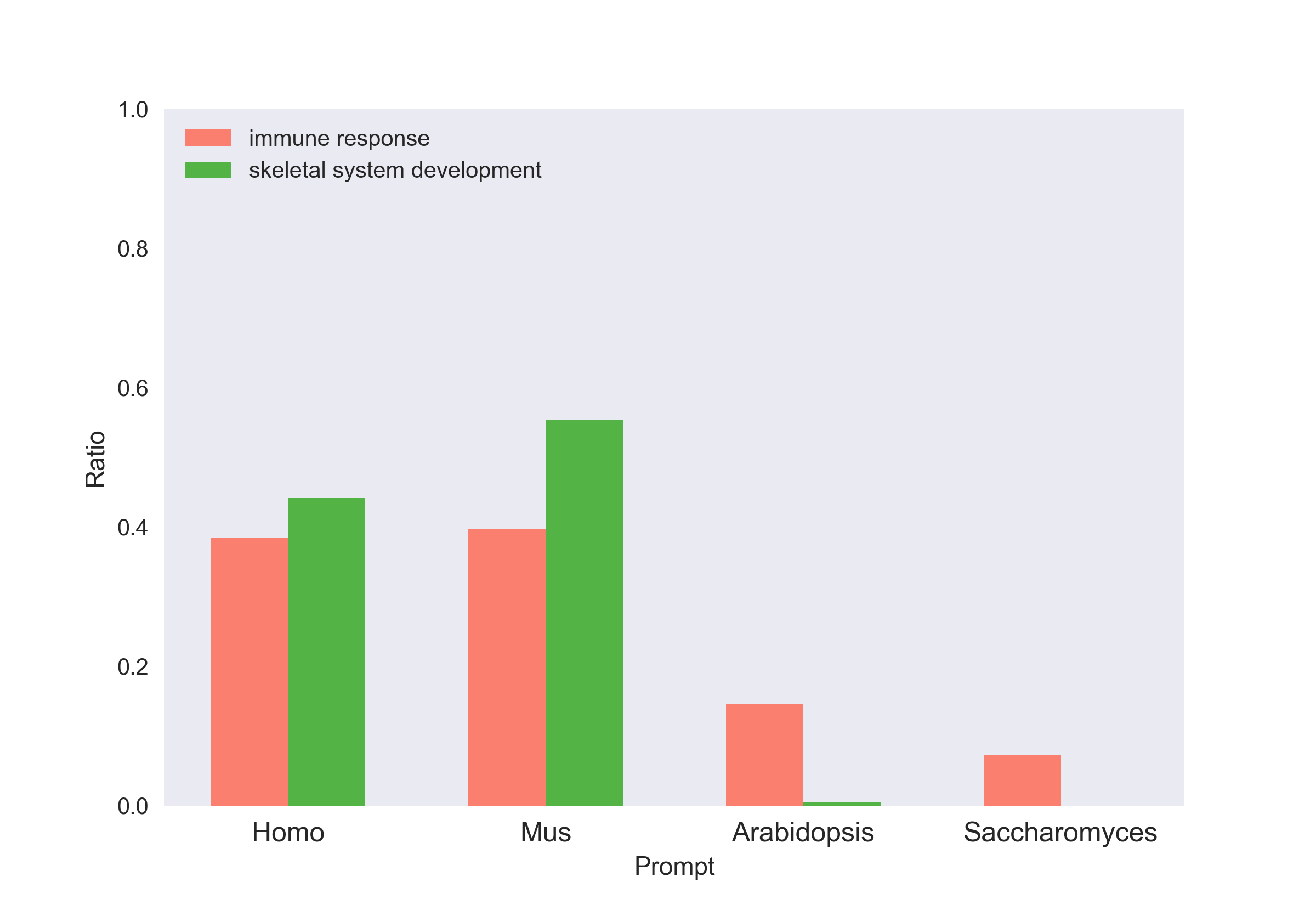


**Supplementary Figure S1** The distribution of function predictions for same set of protein sequences corresponding to the species (prompt) of Homo, Mus, Arabidopsis, and Saccharomyces.
