# Supplementary material for: FAPM: functional annotation of proteins using multimodal models beyond structural modeling
Source: Bioinformatics. 2024 Nov 14;40(12):btae680. doi: 10.1093/bioinformatics/btae680 (PMC11630832; doi:10.1093/bioinformatics/btae680)
Supplement: btae680_Supplementary_Data [file btae680_supplementary_data.zip › Table S1.docx]

| Name | location | Functional labels |
| --- | --- | --- |
| Gp41 | cytosol | DNA binding; protein-protein interaction |
| Gp44 | cytosol | DNA binding |
| Gp45 | cytosol | DNA binding |
| Gp46 | cytosol | Protein-protein interaction |
| Gp49 | cytosol | RNA binding |
| Gp60 | cytosol | Protein-protein interaction |
| Gp33 | cytosol | Protein-protein interaction |
| Gp27 | cytosol | DNA binding |
| Gp35.1 | cytosol | Toxin activity |
| RpbA | cytosol | Protein-protein interaction |
| Exod | cytosol | Dual nuclease activity |
| Gp57B | cytosol | Catalase activity |
| Pin | cytosol | Peptidase inhibitor activity; Protein-protein interaction |
| Mrh | cytosol | DNA binding; Protein-protein interaction |
| Cef | cytosol | RNA binding; Protein-protein interaction |
| MsyB | cytosol | Protein-protein interaction, E. coli |
| YciZ | cytosol | Signal transduction; Protein-protein interaction, B. subtilis |
| YlaB | inner membrane | Signal transduction; Protein-protein interaction, B. subtilis |
| Ysdb | inner membrane | Signal transduction; Protein-protein interaction, B. subtilis |
| RsiW | transmembrane | Signal transduction; Protein-protein interaction, B. subtilis |
| Gp2 | cytosol | Protein-protein interaction |
| Gp6 | cytosol | DNA binding |
| Gp8 | cytosol | DNA binding |
| Gp12 | cytosol | DNA binding |
| ss1 |  | DNA binding |
| sm2 | cytosol | Protein-protein interaction |
| sl1 | cytosol | Protein-protein interaction |
| 75 | cytosol | DNA binding |
| 235 | cytosol | DNA binding |

Supplementary Table S1 the experimentally verified bacteriophage and bacteria protein that are unannotated in UniProt. Proteins are bacteriophage protein unless listed otherwise.
