# Supplementary figures and images for: FAPM: functional annotation of proteins using multimodal models beyond structural modeling
Source: Bioinformatics. 2024 Nov 14;40(12):btae680. doi: 10.1093/bioinformatics/btae680 (PMC11630832; doi:10.1093/bioinformatics/btae680)

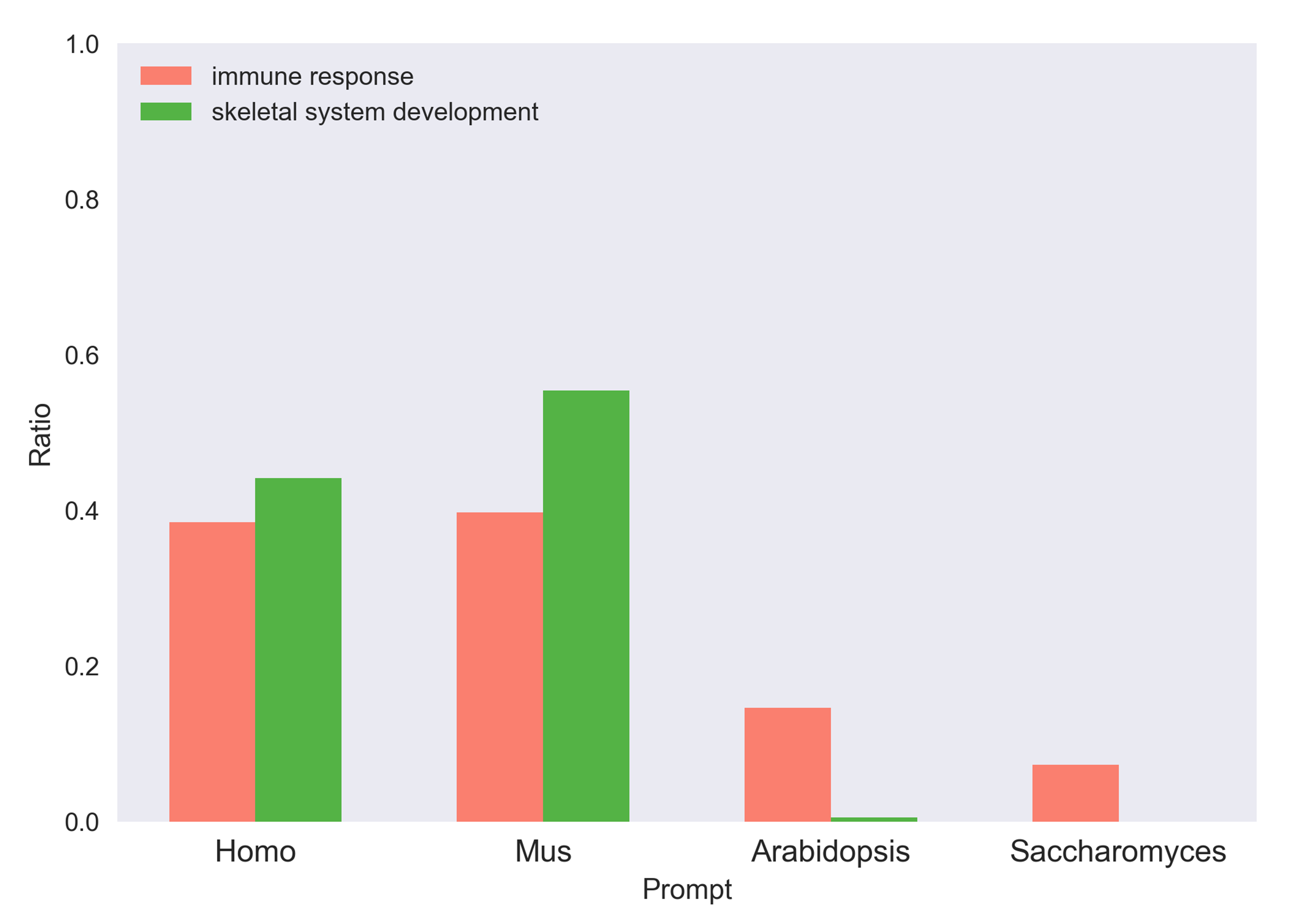

Supplement: btae680_Supplementary_Data [file btae680_supplementary_data.zip › Figure S1.tif]
